# Multimodal Risk-Adapted Treatment in Surgical Patients With Synovial Sarcoma: A Preoperative Nomogram-Guided Adjuvant Treatment Strategy

**DOI:** 10.3389/fsurg.2020.579726

**Published:** 2020-12-21

**Authors:** Ziliang Zeng, Hao Yao, Dongming Lv, Qinglin Jin, Yiying Bian, Yutong Zou, Jian Tu, Bo Wang, Lili Wen, Xianbiao Xie

**Affiliations:** ^1^Department of Musculoskeletal Oncology Center, The First Affiliated Hospital of Sun Yat-sen University, Guangzhou, China; ^2^Guangdong Provincial Key Laboratory of Orthopedics and Traumatology, Guangzhou, China; ^3^State Key Laboratory of Oncology in South China, Department of Anesthesiology, Sun Yat-Sen University Cancer Center, Guangzhou, China

**Keywords:** synovial sarcoma (SS), nomogram, risk assessment, personal treatment, SEER (Surveillance Epidemiology and End Results) database

## Abstract

**Background:** Synovial sarcoma is characterized by heterogeneous clinical manifestations, making it difficult to evaluate individual patients' prognoses and design personal treatment schemes. We established an effective preoperative nomogram to predict cancer-specific survival (CSS) and present a risk-adapted adjuvant treatment strategy in surgical patients with synovial sarcoma.

**Methods:** This retrospective study included patients from the Surveillance, Epidemiology, and End Results (SEER) database who were diagnosed with synovial sarcoma between 1996 and 2015. The patients were randomly divided into training and validation groups. The predictors were selected using univariate and multivariate Cox hazards models. The nomogram performance was verified for its discriminatory ability and calibration. We further stratified the patients into different risk groups according to the nomogram scores and compared the efficacy of chemotherapy, radiotherapy, and combination of radiotherapy and chemotherapy.

**Results:** There were 915 patients enrolled in our study, with 874 patients either alive or dead due to synovial sarcoma. We established a nomogram to predict 5-year CSS based on independent factors, including sex, age, grade, tumor size, location, and extent (all *p* < 0.05). Our model showed a consistently good discriminatory ability and calibration for predicting 5-year CSS in both the training (*c*-index = 0.78, 95% CI 0.75–0.81) and validation (*c*-index = 0.73, 95% CI 0.68–0.78). Based on their nomogram scores, we divided patients into 5 groups. Compared to patients without adjuvant treatment, nomogram I patients with adjuvant treatment had no improvements in 5-year CSS (100.0% vs. 100.0%), nomogram II patients had higher 5-year CSS with radiotherapy or chemotherapy (92.9% vs. 72.2%, *p* = 0.015), nomogram III patients had higher 5-year CSS with combination of chemotherapy and radiotherapy (70.1% vs. 47.2%, *p* = 0.004), nomogram IV patients had higher 5-year CSS with radiotherapy (41.3% vs. 15.6%, *p* = 0.015), and nomogram V patients had no improvements in 5-year CSS rates with adjuvant treatment (28.9% vs. 16.9%, *p* = 0.18).

**Conclusion:** The nomogram showed a satisfactory discriminatory ability and calibration for predicting 5-year CSS in synovial sarcoma patients. Based on this nomogram, we stratified synovial sarcoma patients according to risk levels, which enabled us to provide a useful grouping scheme that can inform multimodal risk-adapted treatment in synovial sarcoma.

## Introduction

Synovial sarcoma is a rare soft tissue sarcoma (STS) characterized by heterogeneous clinical manifestations that can occur at almost any anatomic site and at any age, with various tumor sizes and histological types (1, 2).

The general therapeutic approach for synovial sarcoma is a multimodal protocol that includes surgical excision as the cornerstone of treatment and chemotherapy and radiotherapy as adjuvant treatments, which could further benefit patient survival. This multimodal adjuvant treatment strategy has improved the prognostic outcome in surgical synovial sarcoma patients, but the selection of the appropriate adjuvant treatment with chemotherapy and radiotherapy remains a challenge for clinicians. The present indication of chemotherapy and radiotherapy in synovial sarcoma patients is similar to other STS, which is primarily recommended in patients with incomplete removal of the tumor.

However, various studies have reported different outcomes of chemotherapy and radiotherapy patients, and the concrete applicable population remains controversial. Due to the rarity and heterogeneity of synovial sarcoma, prognostic prediction studies have been limited, with small sample sizes and single-institution designs, and the heterogeneity of the results in previous studies makes it difficult to reach a clinical consensus (3–5).

In the era of precision medicine, nomograms are one of the most widely applied risk assessment tools to evaluate the survival risk for a particular patient and to plan individualized treatment (4). The Surveillance, Epidemiology, and End Results (SEER) database, which covers 28% of the United States population, has become increasingly relevant for studying clinical manifestations and tumor outcomes among rare sarcomas (6, 7). The main purpose of this study was to establish an effective preoperative nomogram to predict 5-year cancer-specific survival (CSS) based on surgical synovial sarcoma patients from the SEER database, thereby evaluating the individual risk in a synovial sarcoma population and exploring risk-adapted adjuvant treatment planning tools for surgical patients with synovial sarcoma.

## Methods

### Patient Population

This retrospective study was based on cases of primary synovial sarcoma between 1996 and 2015 from the SEER database. The case selection criteria were as follows: (1) a diagnosis of synovial sarcoma (International Classification of Diseases for Oncology (ICD-O)-3 codes: 9040/3-9043/3) with positive histological confirmation; (2) first diagnosis between 1996 and 2015 with available follow-up; (3) accepted surgical resection; and (4) complete data, including age, sex, site, histology subtype, differentiation, tumor size, tumor extent, and the American Joint Committee on Cancer (AJCC) stage, as well as the administration of adjuvant treatment, including radiotherapy and chemotherapy. The exclusion criteria were (1) secondary synovial sarcoma or multiple primary tumors; (2) well-differentiated (Grade I); (3) available follow-up time of surviving patients <18 months; and (4) age > 80 years.

### Data Extraction and Synthesis

We extracted the following clinical characteristics: age, sex, primary site, histological subtype, differentiation, tumor size, and the extent of disease (EOD). The EOD was classified as localized (defined as an invasive tumor confined to the tissue of origin), regional (defined as a tumor involving adjacent connective tissue, including bone, cartilage, and major vessel invasion), or distant (defined as further discontinuous extension and distal metastasis at presentation). Sex, primary site, histological subtype, differentiated grade, and EOD were entered as categorical indicators. The continuous variables (age and tumor size) were categorized using restricted cubic splines with 5 knots at the 5, 27.5, 50, 72.5, and 95th percentiles.

The definite endpoint was death in the follow-up timeframe, and the patients' survival status at their last follow-up was censored. Overall survival (OS) was defined as the time from primary diagnosis to death attributed to any cause, and CSS was defined as the time from primary diagnosis to death attributed to synovial sarcoma.

### Nomogram Establishment and Validation

The nomogram was established for the preoperative prediction of 5-year CSS in synovial sarcoma patients. The enrolled CSS patients were randomly divided into a training cohort and a validation cohort at a ratio of 7:3 (set seed 20200726). Patient characteristics were compared between the two groups, using the Chi-squared test for categorical variables and the rank-sum test for ordinal variables. The training cohort was used to establish the nomogram. A univariate Cox analysis was used to screen for prognostic factors based on significant differences in CSS (*p* < 0.05). The screening results were further analyzed as prognostic predictors using a multivariate Cox proportional hazards model.

The eligible predictors were used to establish the nomogram. The model performance was verified in both the resampling training cohort and validation cohort using a calibration plot, time-dependent receiver operating characteristic curve (t-ROC), and concordance index (*c*-index) (8). The calibration plot and a Hosmer–Lemeshow goodness-of-fit test (H–L test)χ were applied to assess the difference between the risk estimated by the nomogram and the observed risk, with confidence intervals (CIs). The t-ROC and *c*-index quantified the discriminatory ability of the nomogram.

### Risk Stratification and Statistical Analysis

Previous studies (9, 10) have demonstrated that nomogram-based scores can be utilized in risk stratification and to guide individual treatment selection. The nomogram scores were continuous variables and were categorized using restricted cubic splines with 5 knots (at the 5, 27.5, 50, 72.5, and 95th percentiles). Continuous demographic data were compared using the rank-sum test, and categorical data were compared using the χ^2^ test. A survival analysis was performed using the Kaplan–Meier (KM) method with the log-rank test. A two-tailed *p* < 0.05 was considered statistically significant. All statistical analyses were conducted with R and SPSS software.

## Results

### Patient Characteristics

From 1996 to 2015, the SEER database contained 1,079 patients with synovial sarcoma who met the inclusion criteria. We excluded 164 patients, and 915 patients were enrolled in our study. The flowchart of the patient selection process, establishment of the nomogram, and risk-adapted therapy strategy is reported in Supplementary Figure 1.

The enrolled patients were followed for a median of 57 months (range: 0–248 months), and survivors were followed for a median of 94 months (range: 18–248 months). A total of 537 (58.7%) patients were still alive at the end of follow-up, and 874 (95.5%) patients were either alive or dead due to synovial sarcoma. The 5-year OS and 5-year CSS rate was 63.0 and 64.9%, respectively.

The median year of diagnosis was 2007 (range: 1996–2015). The annual numbers of newly diagnosis and dead patients are displayed in Supplementary Figure 2, and the year of diagnosis was not a statistically significant factor affecting the 5-year OS or CSS of synovial sarcoma patients during this period (*p* = 0.79 and *p* = 0.91, respectively).

### Independent Prognostic Factors Predicting CSS

The patient characteristics and their therapies are shown in Table 1. Among the eligible CSS patients, 612 were enrolled in the training cohort and 262 were enrolled in the validation cohort.

**Table 1:** Patients clinical and therapeutic manifestation.

**Characteristic**	**Total**	**CSS patients^†^**	**Training cohort**	**Validated cohort**
	**(*n* = 915)**	**(*n* = 874)**	**(*n* = 612)**	**(*n* = 262)**
**Age**				
Median (P_5_-P_95_)	35.0 (13.0–66.0)	34.0 (13.0–65.0)	34.0 (13.0–64.0)	35.0 (14.0–65.9)
**Sex**				
Female	424 (46.3)	406 (46.5)	277 (45.3)	129 (49.2)
Male	491 (53.7)	468(53.5)	335(54.7)	133 (50.8)
**Primary site**				
Extremity	618 (67.5)	598 (68.4)	428 (69.6)	172 (65.6)
Axial	297 (32.5)	276 (31.6)	186 (30.4)	90 (34.4)
**Histological subtype**				
NOS^‡^	327 (35.7)	312 (35.7)	227 (37.1)	85 (32.4)
Monophasic cell	377 (41.2)	360 (41.2)	254 (41.5)	106 (40.5)
Biphasic cell	211 (23.1)	202 (23.1)	131 (21.4)	71 (27.1)
**Grade^§^**				
II	219 (23.9)	209 (23.9)	148 (24.2)	61 (23.3)
III	696 (76.1)	665 (76.1)	464 (75.8)	201 (76.7)
**Tumor size**				
Median (P_5_-P_95_)	6.5 (1.8–17.0)	6.6 (1.7–17.0)	6.4 (1.6–17.0)	7.5 (1.8–17.0)
**Extend**				
Localized	571 (62.4)	545 (62.4)	372 (60.8)	173 (66.0)
Regional	257 (28.1)	247 (28.3)	176 (28.8)	71 (27.1)
Distant	87 (9.5)	82 (9.4)	64 (10.5)	18 (6.9)
**AJCC stage^¶^**				
II stage	306 (33.4)	292 (33.4)	216 (35.3)	76 (29.0)
III stage	507 (55.4)	485 (55.5)	319 (52.1)	166 (34.2)
IV stage	102 (11.1)	97 (11.1)	77 (12.6)	20 (7.6)
**Adjuvant treatment^Ø^**				
No treatment	130 (14.2)	124 (14.2)	87 (14.2)	37 (14.1)
Radio only	347 (37.9)	340 (38.9)	227 (37.1)	113 (43.1)
Chemo only	181 (19.8)	164 (18.8)	113 (18.5)	51 (19.5)
Chemo+Radio	257 (28.1)	246 (28.1)	185 (30.2)	87 (33.0)

† *CSS patients are enrolled patients who were alive or dead attributed to synovial sarcoma*.

‡ *NOS is “none of specific”*.

§ *Grade is WHO pathological grades classification of bone tumor*.

¶ *AJCC stage is assessed with the “The AJCC 8th Edition Staging System for Soft Tissue Sarcoma of the Extremities or Trunk,” “The AJCC 8th Edition Staging System for Soft Tissue Sarcoma of the Retroperitoneum,” and “The AJCC 8th Edition Staging System for Soft Tissue Sarcoma of the Head or Neck.” The III stage includes the IIIA stage and IIIB stage*.

Ø *Adjuvant treatment is additional treatment to patients received surgical resection. Radio stands for radiotherapy, Chemo stand for chemotherapy, Chemo+Radio stands for a combination of chemotherapy and radiotherapy*.

Sex, age, tumor location, differentiation, histological subtype, tumor size, and the EOD were considered as the prognostic factors. The continuous effects of tumor size and age were modeled with restricted cubic splines and indicated that the estimated spline function of the tumor size was “J” shaped, while that of age was relatively linear (non-linear test: age: χ^2^ = 0.37, *p* = 0.83; size: χ^2^ = 13.80, *p* = 0.001) (Supplementary Figure 3). Based on the estimated spline effect, we categorized the continuous indicators using the previously listed percentiles; tumor size was categorized as ≤ 2.1, 2.2–4.2, 4.3–6.0, 6.1–12.5, 12.6–17, or >17 cm, and age was categorized as ≤ 15, 16–40, 41–55, 56–65, 66–70, or >70 years. The results of the univariate and multivariate analyses of prognostic factors for CSS in the training group are shown in Table 2. Male sex, advanced age, higher grade, axial site, larger tumor size, and progressed EOD were independent predictors related to a poor CSS rate (all *p* < 0.05 in both univariate and multivariate analyses; the KM survival curves are shown in Supplementary Figure 4).

**Table 2:** Univariate and multivariate analysis of patients' characteristics with CSS.

**Characteristic**	**Univariate analysis**	**Multivariate analysis**	
	***p*-value**	**HR (95%CI)**	***p*-value**
Histological subtype^†^	0.41		–
Monophasic cell			
Biphasic cell			
NOS			
Age	<0.001^***^	1.02 (1.01–1.03)	<0.001^***^
Tumor size	<0.001^***^	1.07 (1.05–1.09)	<0.001^***^
Sex	0.014		0.012^**^
Female		Reference	
Male		0.72 (0.55–0.93)	
Primary site	0.001^***^		0.001^***^
Extremity		Reference	
Axial		0.62 (0.48–0.82)	
Grade	0.019^**^		0.02^*^
II		Reference	
III		0.66 (0.47–0.94)	
Extend	<0.001^***^		<0.001^***^
Localized		Reference	
Regional		0.23 (0.15–0.33)	
Distant		0.31 (0.21–0.45)	

† *In univariate Cox analysis, histology subtype is not a statistically significant factors relating to CSS rate and it is not enrolled in the multivariate analysis (p = 0.49)*.

* *P < 0.05*;

** *P < 0.01*;

*** *P < 0.001*.

### Establishment and Validation of the Nomogram

**Figure 1:**
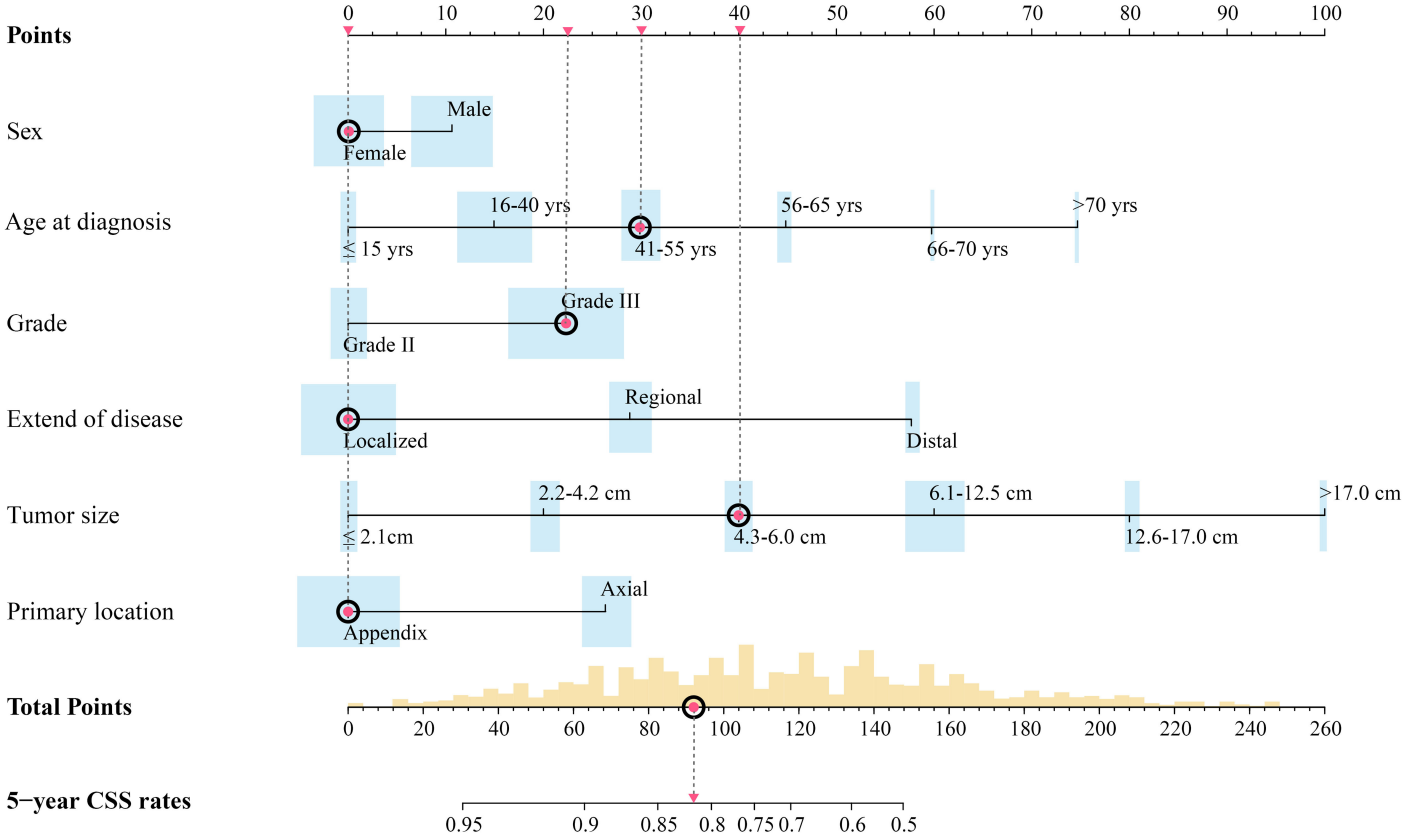
Preoperative nomogram model predicting 5-year CSS rate for surgical patients with synovial sarcoma. Width of bars stands for the population proportion of different characters and different scores. Red spots stand for the hypothetic patient in text: a hypothetical 43-year-old female patient with a primary high-grade SS tumor localized on the extremity with dmax of 5.2 cm.

**Figure 2:**
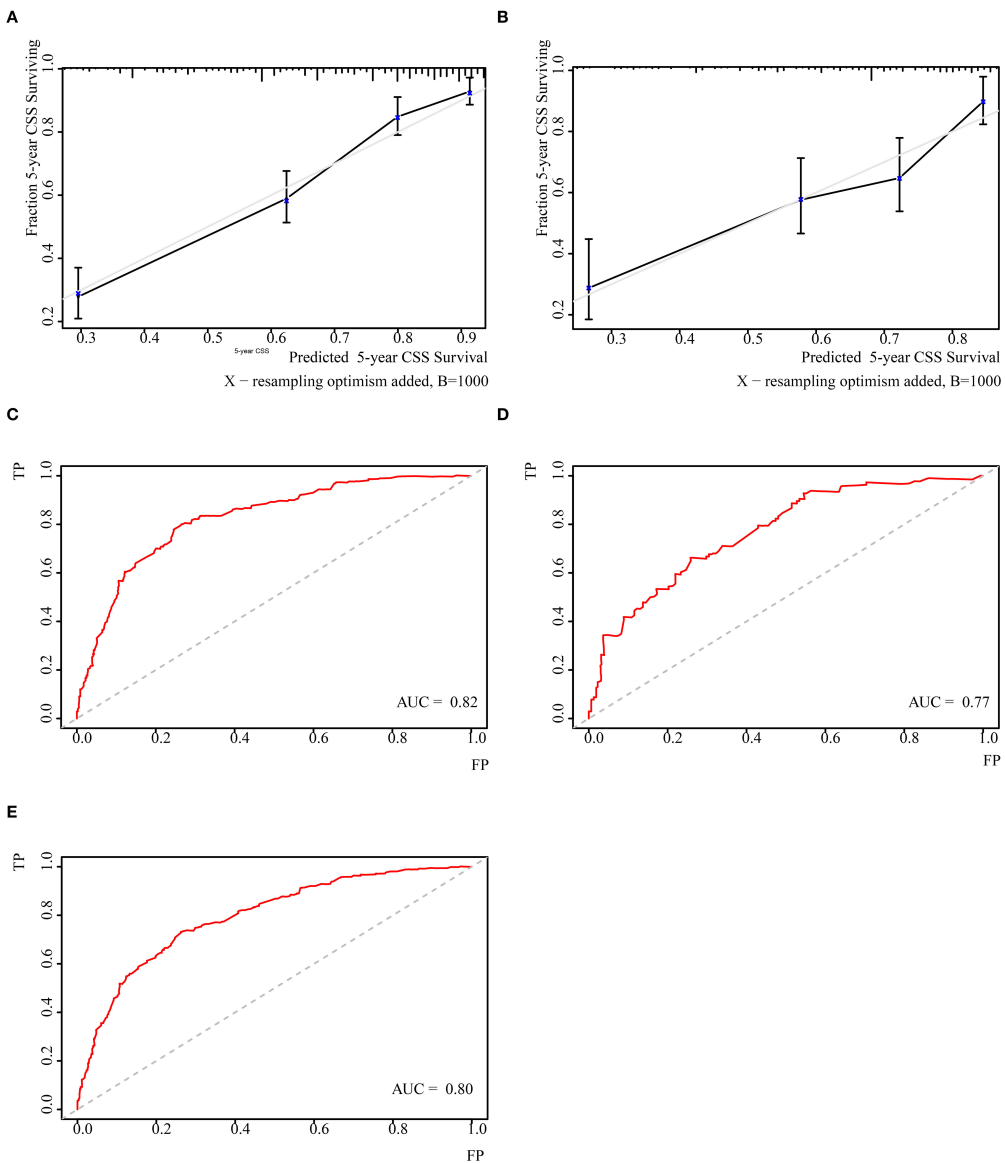
Validation of the nomogram. **(A)** Cablication plot of 5-year CSS in training group; **(C)** Cablication plot of 5-year CSS rate in validating group; **(B)** t-ROC plot of 5-year CSS rate in training group; **(D)** t-ROC plot of 5-year CSS rate in validating group; **(E)** t-ROC plot of 5-year OS rate in total population.

Independent prognostic factors were used to establish the nomogram for predicting 5-year CSS. The nomogram model is shown in Figure 1, and the validation plots are shown in Figure 2. The model showed a good discriminatory ability in the internal validation assessment (area under the curve (AUC) = 0.82, *c*-index = 0.78, 95% CI = 0.75–0.81) and good calibration for predicting 5-year CSS (H-L test: χ^2^ = 14.29, *p* = 0.11).

The nomogram underwent further external validation, in which the nomogram showed a good discriminatory ability (AUC = 0.77, *c*-index = 0.73, 95% CI = 0.68–0.78) and calibration for the prediction of 5-year CSS (H-L test: χ^2^ = 3.35, *p* = 0.95). This result suggested that the nomogram had a consistently reliable ability to predict CSS in the training and validation groups.

The nomogram was also tested for its predictive ability among the total synovial sarcoma patient population. Based on the nomogram, we calculated a personal score for the total population (median 112.19, range: 0.00–246.84). The nomogram showed a comparable discriminatory ability (AUC=0.80) and goodness of fit for survival (H–L test: χ^2^ = 10.25, *p* = 0.33) among the total population compared to the CSS population. This finding indicates that the model had an extended predictive ability among the total synovial sarcoma population and could have a stable ability to predict survival among synovial sarcoma patients.

### Risk Stratification and Corresponding Clinical Features

**Figure 3:**
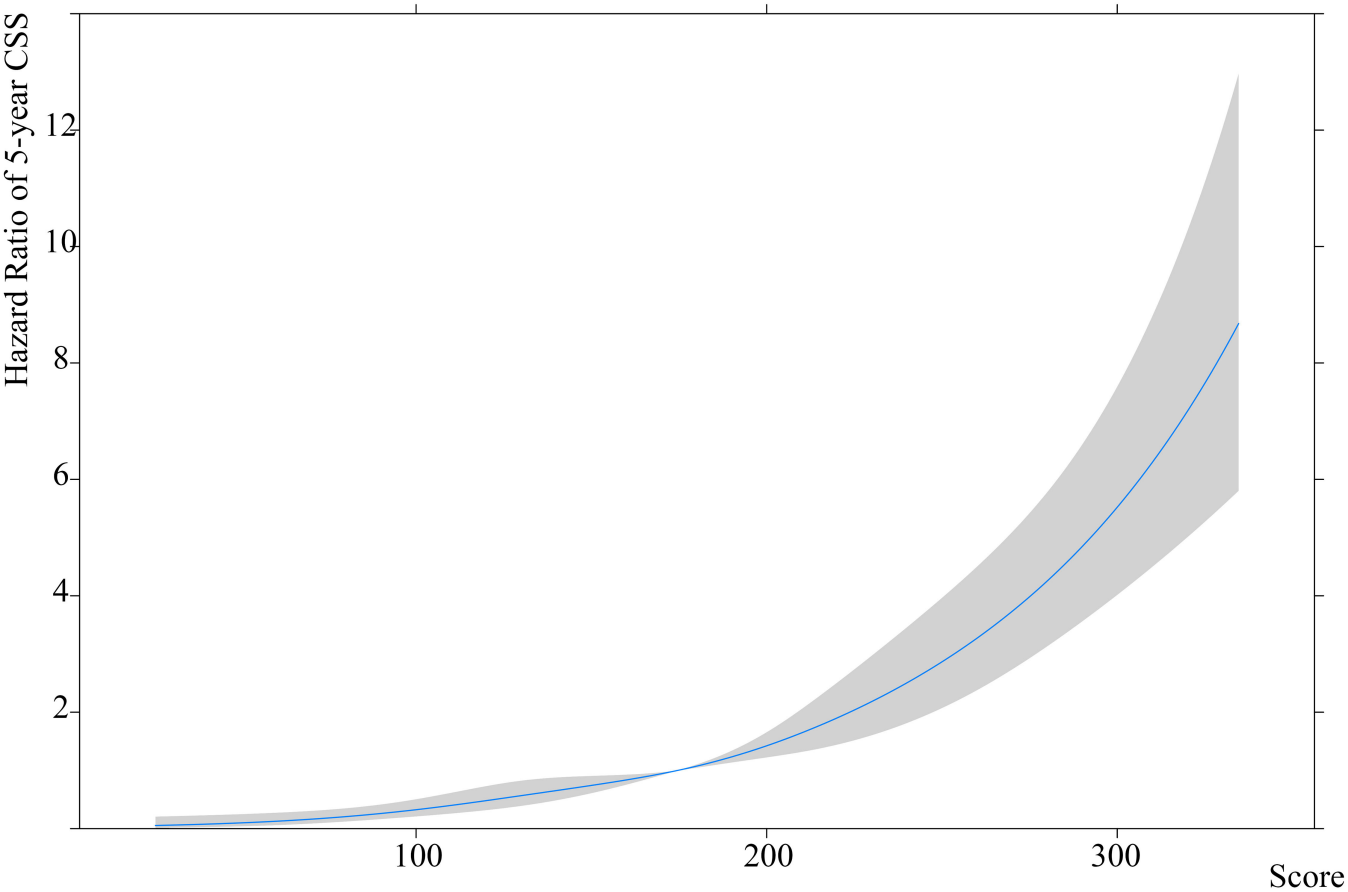
The estimated spline function of the nomogram scores on a 5-year CSS rate.

The CSS patients were used to establish the nomogram-based risk stratification. The estimated spline function of the nomogram score on CSS was J shaped, with the lowest hazard being 20.0 and the highest being 225.9. The 5, 27.5, 50, 72.5, and 95th percentiles were 85.2, 157.6, 208.4, and 222.8, respectively (non-linear test: χ^2^ = 1.96, *p* = 0.58) (Figure 3).

Using the 5 percentiles, the nomogram score was categorized into 7 scoring intervals and both the 5-year OS rate and 5-year CSS rate decreased as the scoring increased in synovial sarcoma patients (5-year OS: ≤ 20.0: 100.0%, 20.1–85.2: 89.2%, 85.3–157.6: 61.4%, 157.7–208.4: 28.9%, 208.5–222.8: 27.7%, 222.9–225.9: 7.8%, and > 225.9: 12.5; 5-year CSS: ≤ 20.0: 100.0%, 20.1–85.2: 91.3%, 85.3–157.6: 62.7%, 157.7–208.4: 30.7%, 208.5–222.8: 28.6%, 222.9–225.9: 8.3%, and > 225.9: 15.4%) (Supplementary Figure 4). Patients with a nomogram score of 157.7–222.8 had comparable survival rates and were included in the same intervals (5-year OS: 28.9% vs. 27.7%, *p* = 0.14; 5-year CSS: 30.7% vs. 27.7%, *p* = 0.12). Patients with a nomogram score >222.8 had the same survival rate and were included in the same intervals (5-year OS: 7.8% vs. 12.5%, *p* = 0.36; 5-year CSS: 8.3% vs. 15.4%, *p* = 0.64).

Their clinical features and treatment protocols in each scoring intervals are shown in Table 3. We observed that the proportions of males and patients with advanced age, high grade, large tumor size, axial location, and progressive disease increased among the higher risk groups (all *p* < 0.05).

**Table 3:** Patients characters and therapy in different risk group.

**Characteristic**	**Nomogram I**	**Nomogram II**	**Nomogram III**	**Nomogram IV**	**Nomogram V**	***p*-value**
	**(*n* = 12)**	**(*n* = 251)**	**(*n* = 480)**	**(*n* = 140)**	**(*n* = 32)**	
Age						<0.001^***^
Median (P_5_-P_95_)	15.5 (9.0–28.5)	28.0 (9.6–53.0)	36.0 (15.0–63.0)	44.0 (17.1–72.0)	58.0 (20.0–77.1)	
Sex						<0.001^***^
Female	12 (100.0)	141 (56.2)	219 (45.6)	44 (31.4)	8 (25.0)	
Male	0 (0.0)	110 (43.8)	261 (54.4)	96 (68.6)	24 (75.0)	
Primary site						<0.001^***^
Extremity	12 (100.0)	226 (90.0)	316 (65.8)	54 (38.6)	10 (31.3)	
Axial	0 (0.0)	25 (10.0)	164 (34.2)	86 (61.4)	22 (68.8)	
Grade					<0.001^***^	
II	12 (100.0)	111 (44.2)	80 (16.7)	15 (10.7)	1 (3.1)	
III	0 (0.0)	140 (55.8)	400 (83.3)	125 (89.3)	31 (96.9)	
Tumor size						<0.001^***^
Median (P_5_-P_95_)	2.0 (0.5–3.9)	3.4 (1.0–7.9)	7.5 (2.8–15.0)	10.2 (5.2–25.4)	16.0 (6.2–36.8)	
Extend						<0.001^***^
Localized	12 (100.0)	228 (90.8)	313 (65.2)	17 (12.1)	1 (3.1)	
Regional	0 (0.0)	23 (9.2)	145 (30.2)	81 (57.9)	8 (25.0)	
Distant	0 (0.0)	0 (0.0)	22 (4.6)	42 (30.0)	23 (71.9)	
AJCC stage^†^						<0.001^***^
II stage	12 (100.0)	199 (79.3)	92 (19.2)	3 (2.1)	0 (0.0)	
III stage	0 (0.0)	52 (20.7)	353 (73.5)	94 (67.1)	8 (25.0)	
IV stage	0 (0.0)	0 (0.0)	35 (7.3)	42 (30.7)	24 (75.0)	
Adjuvant treatment						<0.001^***^
No treatment	1 (8.3)	22 (8.8)	67 (14.0)	30 (21.4)	10 (31.3)	
Radio only	5 (41.7)	57 (22.7)	87 (18.1)	23 (16.4)	9 (28.1)	
Chemo only	3 (25.0)	72 (28.7)	211 (44.0)	51 (36.4)	10 (31.3)	
Radio+Chemo	3 (25.0)	100 (39.8)	115 (24.0)	36 (25.7)	3 (9.4)	

† *AJCC stage is assessed with the “The AJCC 8th Edition Staging System for Soft Tissue Sarcoma of the Extremities or Trunk,” “The AJCC 8th Edition Staging System for Soft Tissue Sarcoma of the Retroperitoneum,” and “The AJCC 8th Edition Staging System for Soft Tissue Sarcoma of the Head or Neck.” The III stage includes the IIIA stage and IIIB stage*.

*** *P < 0.001*.

Compared to the AJCC stage, our model adjusted and redistributed the population into 5 risk stages based on synovial sarcoma-specific factors. Patients with a nomogram score ≤ 20.0 were all categorized as AJCC stage II (100.0%), and these patients were entered as nomogram stage I. Patients with a score of 20.1–85.2 were categorized as AJCC stage II (79.3%) and III (20.7%), and these patients were entered as nomogram stage II. Patients with a score of 85.3–157.6 were mainly categorized as AJCC stage II (19.2%) and III (73.5%), and these patients were entered as nomogram stage III. Patients with a score of 157.7–222.8 or >222.8 were categorized as AJCC stage III (59.3%) and IV (39.0%), and these patients were entered as nomogram stage IV and V, respectively.

### Risk-Adapted Therapy Using Nomogram-Based Risk Stratification

Overall, surgical patients who underwent adjuvant treatment with radiotherapy, chemotherapy, or a combination of radiotherapy and chemotherapy had higher 5-year OS and CSS rates than patients without adjuvant treatment (radiotherapy vs. no adjuvant treatment: 5-year OS 65.2% vs. 41.8%, *p* < 0.001; 5-year CSS 66.1% vs. 43.1%, *p* < 0.001; chemotherapy vs. no adjuvant treatment: 5-year OS 61.4% vs. 41.8%, *p* = 0.01; 5-year CSS 65.7% vs. 43.1%, *p* = 0.003; combination of radiotherapy and chemotherapy vs. no adjuvant treatment: 5-year OS 71.6% vs. 41.8%, *p* < 0.001; 5-year CSS 73.5% vs. 43.1%, *p* < 0.001). Patients who received only radiotherapy showed no significant differences in 5-year OS or CSS compared to those who received only chemotherapy (5-year OS 65.2% vs. 61.4%, *p* = 0.47; 5-year CSS 66.1% vs. 65.7%, *p* = 0.91). Patients with a combination of radiotherapy and chemotherapy had significantly higher 5-year OS and CSS compared to patients who received only radiotherapy or only chemotherapy (combination of radiotherapy and chemotherapy vs. radiotherapy only: 5-year OS 71.6% vs. 65.2%, *p* = 0.003; 5-year CSS 73.5% vs. 66.1%, *p* = 0.001; combination of radiotherapy and chemotherapy vs. chemotherapy only: 5-year OS 71.6% vs. 61.4%, *p* = 0.002; 5-year CSS 73.5% vs. 65.7%, *p* = 0.01).

Among nomogram stage I patients, surgical patients who received adjuvant treatment with radiotherapy, chemotherapy, or a combination of radiotherapy and chemotherapy had no significant differences in 5-year OS or CSS rates compared to patients without adjuvant treatment (radiotherapy vs. no adjuvant treatment: 5-year OS 100.0% vs. 100.0%; 5-year CSS 100.0% vs. 100.0%; chemotherapy vs. no adjuvant treatment: 5-year OS 100.0% vs. 100.0%; 5-year CSS 100.0% vs. 100.0%; combination of radiotherapy and chemotherapy vs. no adjuvant treatment: 5-year OS 100.0% vs. 100.0%; 5-year CSS 100.0% vs. 100.0%).

**Figure 4:**
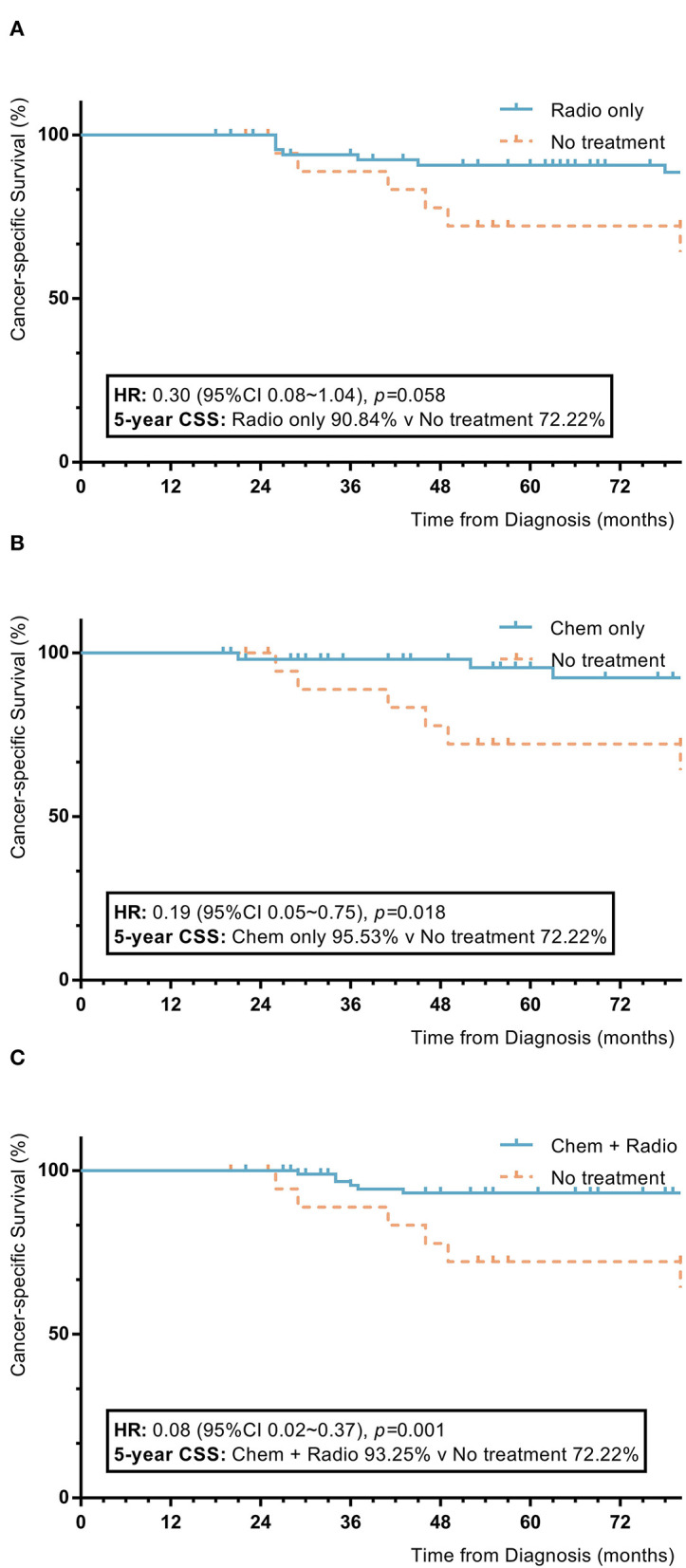
KM plot showing the difference of CSS among nomogram stage II synovial sarcoma patients with different adjuvant treatment. Adjuvant treatment is additional treatment to patients received surgical resection. Radio, radiotherapy; Chemo, Chemotherapy; Chemo+Radio, a combination of chemotherapy and radiotherapy. **(A)** Between radiotherapy and no adjuvant treatment; **(B)** Between chemotherapy and no adjuvant treatment; **(C)** Between a combination of radiotherapy and chemotherapy and no adjuvant treatment.

Among nomogram stage II patients, surgical patients who received adjuvant treatment with only radiotherapy or chemotherapy had higher 5-year OS and CSS rates to those of patients without adjuvant treatment (radiotherapy vs. no adjuvant treatment: 5-year OS 89.4% vs. 73.7%, *p* = 0.04; 5-year CSS 90.8% vs. 72.2%, *p* = 0.058; chemotherapy vs. no adjuvant treatment: 5-year OS 91.3% vs. 73.7%, *p* = 0.03; 5-year CSS 95.5% vs. 72.2%, *p* = 0.018). Patients with a combination of radiotherapy and chemotherapy also showed higher 5-year OS and CSS rates than patients without adjuvant treatment (combination of radiotherapy and chemotherapy vs. no adjuvant treatment: 5-year OS 91.1% vs. 73.7%, *p* = 0.001; 5-year CSS 93.2% vs. 72.2%, *p* = 0.001). Patients who received only radiotherapy or chemotherapy had 5-year OS and CSS rates comparable to those of patients with a combination of radiotherapy and chemotherapy (radiotherapy vs. combination of radiotherapy and chemotherapy: 5-year OS 89.4% vs. 91.1%, *p* = 0.37; 5-year CSS 93.2% vs. 90.8%, *p* = 0.21; chemotherapy vs. combination of radiotherapy and chemotherapy: 5-year OS 91.3% vs. 91.1%, *p* = 0.58; 5-year CSS 93.2% vs. 95.5%, *p* = 0.70). Patients who received only radiotherapy had 5-year OS and CSS rates comparable to those of patients who received only chemotherapy (radiotherapy vs. chemotherapy: 5-year OS 89.4% vs. 91.3%, *p* = 0.86; 5-year CSS 90.8% vs. 93.2%, *p* = 0.57) (Figure 4).

**Figure 5:**
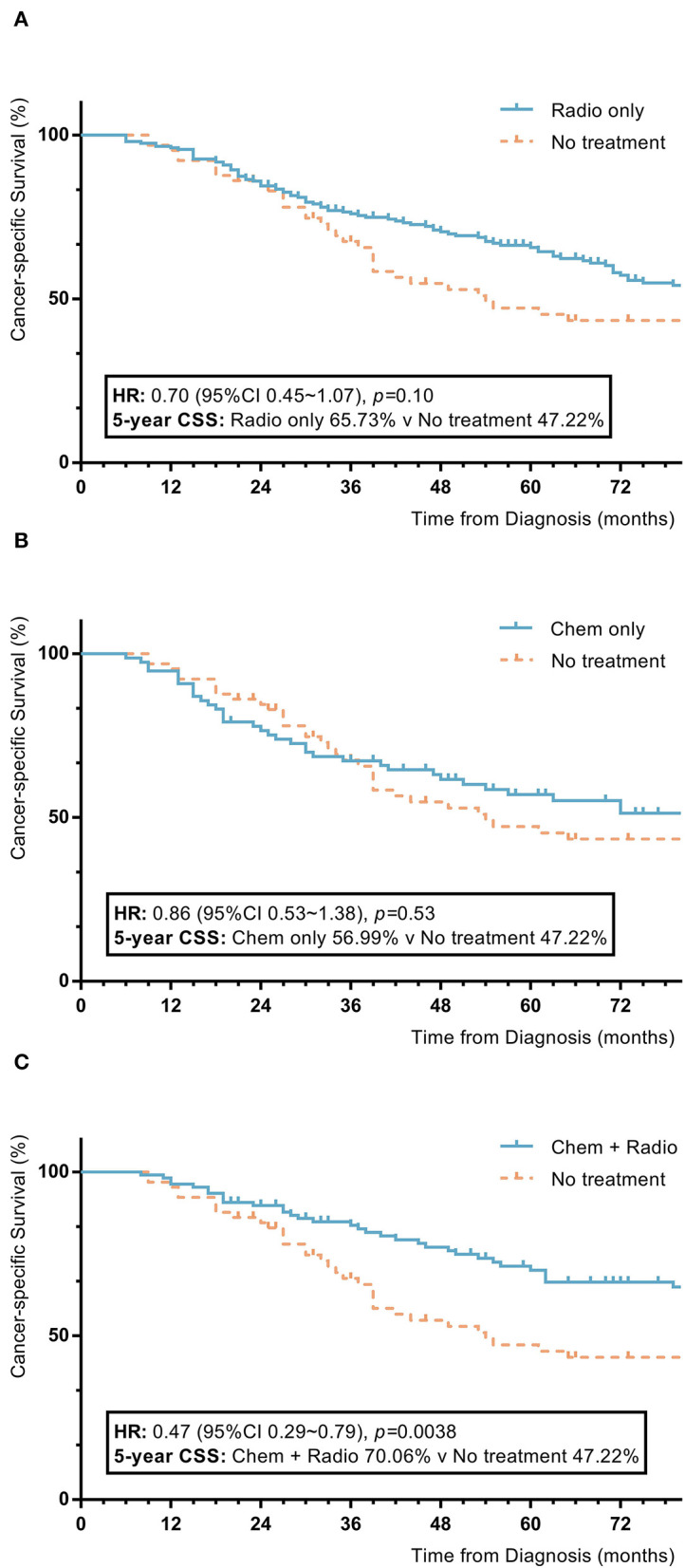
KM plot showing the difference of CSS among nomogram stage III synovial sarcoma patients with different adjuvant treatment. Adjuvant treatment is additional treatment to patients received surgical resection. Radio, radiotherapy; Chemo, chemotherapy; Chemo+Radio, a combination of chemotherapy and radiotherapy. **(A)** Between radiotherapy and no adjuvant treatment; **(B)** Between chemotherapy and no adjuvant treatment; **(C)** Between a combination of radiotherapy and chemotherapy and no adjuvant treatment.

Among nomogram stage III patients, those who received only chemotherapy or radiotherapy did not have significant improvements in 5-year OS or CSS rates compared to patients without adjuvant treatment (radiotherapy vs. no adjuvant treatment: 5-year OS 65.2% vs. 45.8%, *p* = 0.07; 5-year CSS 65.7% vs. 47.2%, *p* = 0.10; chemotherapy vs. no adjuvant treatment: 5-year OS 55.1% vs. 45.8%, *p* = 0.72; 5-year CSS 57.0% vs. 47.2%, *p* = 0.53). Patients with a combination of radiotherapy and chemotherapy had significantly higher 5-year OS and CSS rates compared to patients without adjuvant treatment (combination of radiotherapy and chemotherapy vs. no adjuvant treatment: 5-year OS 70.4% vs. 45.8%, *p* = 0.006; 5-year CSS 70.1% vs. 47.2%, *p* = 0.004) (Figure 5).

**Figure 6:**
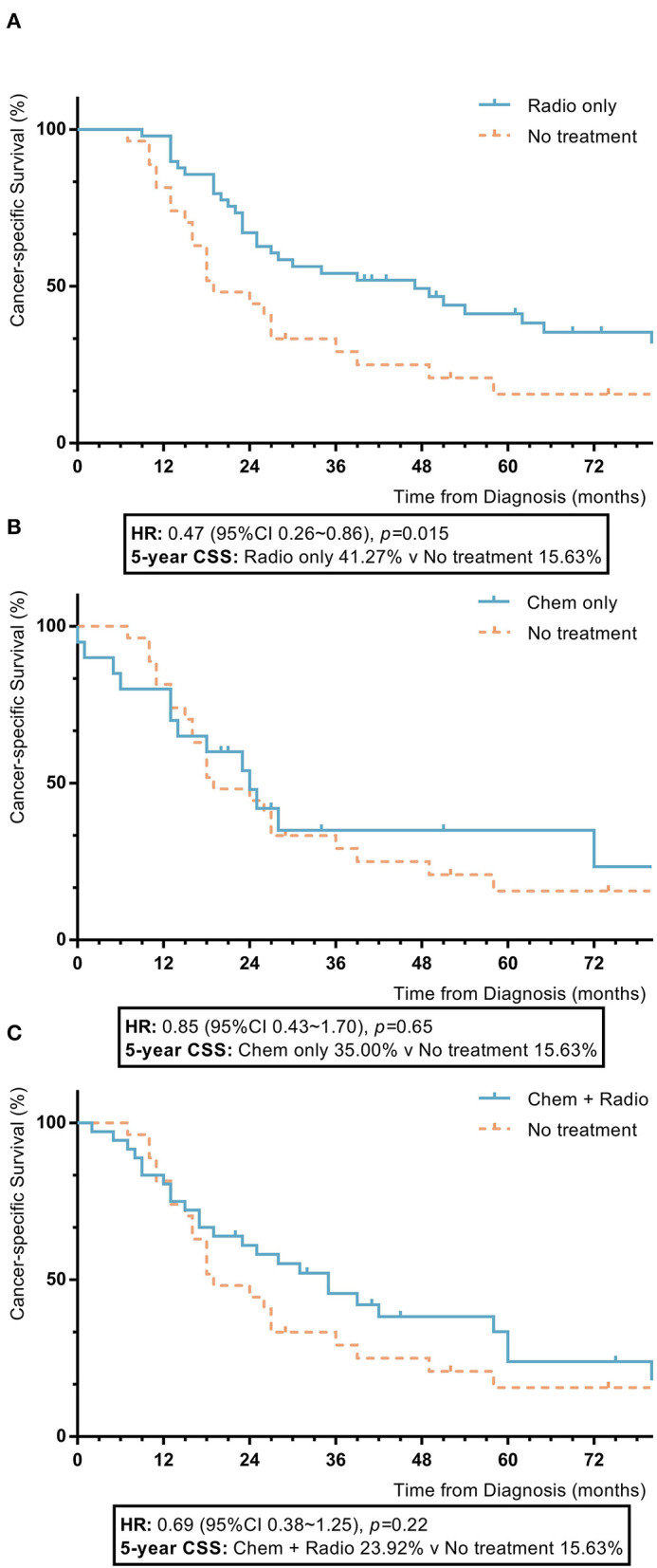
KM plot showing the difference of CSS among nomogram stage IV synovial sarcoma patients with different adjuvant treatments. Adjuvant treatment is additional treatment to patients received surgical resection. Radio, radiotherapy; Chemo, chemotherapy; Chemo+Radio, a combination of chemotherapy and radiotherapy **(A)** Between radiotherapy and no adjuvant treatment; **(B)** Between chemotherapy and no adjuvant treatment; **(C)** Between a combination of radiotherapy and chemotherapy and no adjuvant treatment.

Among nomogram stage IV patients, patients with radiotherapy had statistically higher 5-year OS and CSS rates compared to patients without adjuvant treatment (radiotherapy vs. no adjuvant treatment: 5-year OS 41.3% vs. 13.8%, *p* = 0.008; 5-year CSS 41.3% vs. 15.6%, *p* = 0.015). Those with chemotherapy and a combination of radiotherapy and chemotherapy had no significant differences in 5-year OS or CSS rates compared to patients who did not receive adjuvant treatment (chemotherapy vs. no adjuvant treatment: 5-year OS 28.2% vs. 13.8%, *p* = 0.83; 5-year CSS 35.0% vs. 15.3%, *p* = 0.65; combination of radiotherapy and chemotherapy vs. no adjuvant treatment: 5-year OS 23.9% vs. 13.8%, *p* = 0.18; 5-year CSS 23.9% vs. 15.6%, *p* = 0.22) (Figure 6).

**Figure 7:**
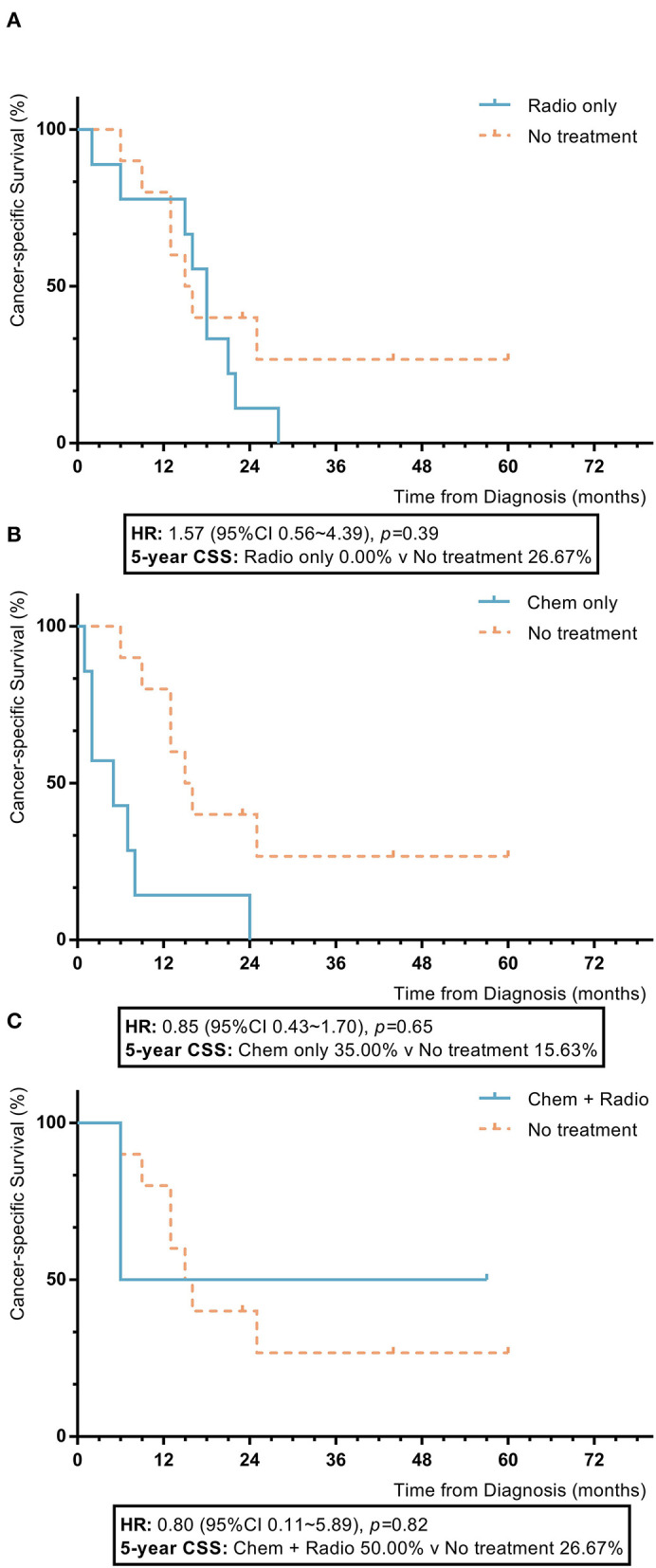
KM plot showing the difference of CSS among nomogram stage V synovial sarcoma patients with different adjuvant treatment. Adjuvant treatment is additional treatment to patients received surgical resection. Radio, radiotherapy; Chemo, chemotherapy; Chemo+Radio, a combination of chemotherapy and radiotherapy. **(A)** Between radiotherapy and no adjuvant treatment; **(B)** Between chemotherapy and no adjuvant treatment; **(C)** Between a combination of radiotherapy and chemotherapy and no adjuvant treatment.

Among nomogram stage V patients, those with adjuvant treatment, including radiotherapy, chemotherapy, and a combination of radiotherapy and chemotherapy, also had no statistically significant improvement in 5-year OS or CSS rates compared to patients who did not receive adjuvant treatment, who had chemotherapy which showed inferior 5-year OS and CSS to those without adjuvant treatment (radiotherapy vs. no adjuvant treatment: 5-year OS 0.0% vs. 26.7%, *p* = 0.32; 5-year CSS 32.7% vs. 16.9%, *p* = 0.24; chemotherapy vs. no adjuvant treatment: 5-year OS 0.0% vs. 26.7%, *p* = 0.002; 5-year CSS 20.0% vs. 16.9%, *p* = 0.20; combination of radiotherapy and chemotherapy vs. no adjuvant treatment: 5-year OS 33.3% vs. 26.7%, *p* = 0.90; 5-year CSS 30.0% vs. 16.9%, *p* = 0.74) (Figure 7).

### Risk-Adapted Therapy Using AJCC Stage

Among AJCC stage II patients, surgical patients who received adjuvant treatment with only radiotherapy or chemotherapy had 5-year OS and CSS rates similar to those of patients without adjuvant treatment (radiotherapy vs. no adjuvant treatment: 5-year OS 85.7% vs. 71.2%, *p* = 0.23; 5-year CSS 87.0% vs. 69.9%, *p* = 0.26; chemotherapy vs. no adjuvant treatment: 5-year OS 84.2% vs. 71.2%, *p* = 0.24; 5-year CSS 88.5% vs. 69.9%, *p* = 0.14). Patients with a combination of radiotherapy and chemotherapy had higher 5-year OS and CSS rates compared to patients without adjuvant treatment (combination of radiotherapy and chemotherapy vs. no adjuvant treatment: 5-year OS 87.8% vs. 71.2%, *p* = 0.009; 5-year CSS 89.1% vs. 69.9%, *p* = 0.005).

Among AJCC stage III patients, those with only radiotherapy or a combination of radiotherapy and chemotherapy had higher 5-year OS and CSS rates compared to patients without adjuvant treatment (radiotherapy vs. no adjuvant treatment: 5-year OS 67.4% vs. 41.3%, *p* = 0.001; 5-year CSS 68.3% vs. 44.0%, *p* = 0.004; combination of radiotherapy and chemotherapy vs. no adjuvant treatment: 5-year OS 58.7% vs. 41.3%, *p* = 0.02; 5-year CSS 60.7% vs. 44.0%, *p* = 0.03). Patients who received only radiotherapy had 5-year OS and CSS rates comparable to those of patients with a combination of radiotherapy and chemotherapy (5-year OS 67.4% vs. 58.7%, *p* = 0.44; 5-year CSS 68.3% vs. 60.7%, *p* = 0.60). Patients who received only chemotherapy had 5-year OS and CSS rates similar to those of patients without adjuvant treatment (5-year OS 43.8% vs. 41.3%, *p* = 0.74; 5-year CSS 46.6% vs. 44.0%, *p* = 0.84).

Among AJCC stage IV patients, those with adjuvant treatment, including radiotherapy, chemotherapy, and a combination of radiotherapy and chemotherapy, had no significant differences in 5-year OS or CSS rates compared to patients who did not receive adjuvant treatment (radiotherapy vs. no adjuvant treatment: 5-year OS 24.9% vs. 16.1%, *p* = 0.56; 5-year CSS 25.5% vs. 16.7%, *p* = 0.50; chemotherapy vs. no adjuvant treatment: 5-year OS 0.0% vs. 16.1%, *p* = 0.054; 5-year CSS 13.3% vs. 16.7%, *p* = 0.21; combination of radiotherapy and chemotherapy vs. no adjuvant treatment: 5-year OS 13.3% vs. 16.1%, *p* = 0.83; 5-year CSS 16.7% vs. 16.7%, *p* = 0.55).

## Discussion

Synovial sarcoma is known for its heterogeneous clinical manifestations, and the decision regarding suitable adjuvant treatment for surgical patients remains a challenge (3–5). Similar to other STS, surgical resection is the cornerstone of treatment for synovial sarcoma, and adjuvant treatment with chemotherapy and radiotherapy could further improve patient survival. However, among the current research, various studies have demonstrated different populations for radiotherapeutic and chemotherapeutic benefits, and a consensus has yet to be reached on the detailed indications. The SEER database is one the largest databases used for STS research, and it provides adequate and high-quality follow-up data that can be used to establish effective nomograms (7). Our study enrolled 915 patients (874 patients either alive or dead due to synovial sarcoma) from the SEER database to establish a preoperative risk-adapted adjuvant therapeutic strategy for synovial sarcoma patients (8, 11).

Multiple studies have identified a number of preoperative prognostic factors in patients with synovial sarcoma, to include sex, age, histological subtype, differentiation, tumor location, tumor size, and the EOD (12–23). These clinical manifestations were included in the candidate prognostic factors for the current model. The Cox analysis identified elderly age, poor differentiation, tumor located in the axial region, larger tumor size, and regional to distant EOD as independent prognostic factors that were negatively associated with 5-year CSS (all *p* < 0.05 in the Cox regression). Based on the enrolled factors, we established a preoperative nomogram for the prediction of 5-year CSS in patients with synovial sarcoma, and the model showed a satisfactory ability to predict CSS in terms of both discrimination and calibration (*c*-index = 0.78, 95% CI = 0.75–0.81, H-L test: χ^2^= 14.29, *p* = 0.11). Compared with synovial sarcoma-specific nomogram models, our study carries the advantage of covering the most preoperative variables identified in previous models, as well as a larger sample size compared with previous reports (3, 5). Our nomogram provides a complete and comprehensive preoperative assessment of risk in patients with synovial sarcoma and facilitates decision-making regarding treatment strategies.

It has been demonstrated that a risk-adapted treatment strategy that combines the proper administration of adjuvant treatment with radiotherapy, chemotherapy, or a combination of radiotherapy and chemotherapy based on the risk level could improve the survival rate in surgical patients with synovial sarcoma (1, 24–26). Based on their nomogram scores and estimated spline function on 5-year CSS, we divided the enrolled surgical patients into 5 risk intervals (nomogram I: ≤ 20.0, nomogram II: 20.1–85.2, nomogram III: 85.3–157.6, nomogram IV: 157.7–222.8, and nomogram V: >222.8) and explored the multimodal risk-adapted treatment of patients with synovial sarcoma.

Our results showed that nomogram stage I patients had a 100% 5-year CSS rate with surgical resection, and additional treatment with radiotherapy and chemotherapy could not further improve their 5-year OS or CSS (radiotherapy vs. no adjuvant treatment: 5-year OS 100.0% vs. 100.0%; 5-year CSS 100.0% vs. 100.0%; chemotherapy vs. no adjuvant treatment: 5-year OS 100.0% vs. 100.0%; 5-year CSS 100.0% vs. 100.0%; combination of radiotherapy and chemotherapy vs. no adjuvant treatment: 5-year OS 100.0% vs. 100.0%; 5-year CSS 100.0% vs. 100.0%). The clinical characteristics of nomogram stage I patients showed a low risk in survival and were consistent with the characteristics of the surgery-only population in Ferrari's report, including primary tumor location in an extremity, small tumor size, and adequate resection on young patients (25).

With surgical resection of the tumor, adjuvant radiotherapy and chemotherapy are alternatively recommended as a local and systemic therapy for synovial sarcoma patients, which is primarily applied with patients at high risk of incomplete removal of the tumor (1, 27, 28). Age, tumor location, the presence of distal metastasis, and histological response are important preoperative prognostic variables in high-grade synovial sarcoma patients undergoing adjuvant treatment (29). As the current results showed, nomogram stage II patients who received only radiotherapy or chemotherapy had higher 5-year OS and CSS rates compared to patients who did not receive adjuvant treatment (radiotherapy vs. no adjuvant treatment: 5-year OS 89.4% vs. 73.7%, *p* = 0.04; 5-year CSS 90.8% vs. 72.2%, *p* = 0.058; chemotherapy vs. no adjuvant treatment: 5-year OS 91.3% vs. 73.7%, *p* = 0.03; 5-year CSS 95.5% vs. 72.2%, *p* = 0.018), and their rates were similar to those of patients who received a combination of both treatments (radiotherapy vs. combination of radiotherapy and chemotherapy: 5-year OS 89.4% vs. 91.1%, *p* = 0.37; 5-year CSS 93.2% vs. 90.8%, *p* = 0.21; chemotherapy vs. combination of radiotherapy and chemotherapy: 5-year OS 91.3% vs. 91.1%, *p* = 0.58; 5-year CSS 93.2% vs. 95.5%, *p* = 0.70). Nomogram stage III patients who received a combination of chemotherapy and radiotherapy showed higher 5-year OS and CSS rates compared to patients without adjuvant treatment (combination of radiotherapy and chemotherapy vs. no adjuvant treatment: 5-year OS 70.4% vs. 45.8%, *p* = 0.006; 5-year CSS 70.1% vs. 47.2%, *p* = 0.004). In nomogram stage IV patients, those received radiotherapy had significantly higher 5-year OS and CSS rates compared to patients without adjuvant treatment (radiotherapy vs. no adjuvant treatment: 5-year OS 41.3% vs. 13.8%, *p* = 0.008; 5-year CSS 41.3% vs. 15.6%, *p* = 0.015). Nomogram V patients who received adjuvant treatment with radiotherapy, chemotherapy, or a combination of radiotherapy and chemotherapy had no significant improvement in 5-year OS or CSS rates compared to patients without adjuvant treatment (radiotherapy vs. no adjuvant treatment: 5-year OS 0.0% vs. 26.7%, *p* = 0.32; 5-year CSS 32.7% vs. 16.9%, *p* = 0.24; chemotherapy vs. no adjuvant treatment: 5-year OS 0.0% vs. 26.7%, *p* = 0.002; 5-year CSS 20.0% vs. 16.9%, *p* = 0.20; combination of radiotherapy and chemotherapy vs. no adjuvant treatment: 5-year OS 33.3% vs. 26.7%, *p* = 0.90; 5-year CSS 30.0% vs. 16.9%, *p* = 0.74). The indications and beneficiaries among different adjuvant treatments on surgical synovial sarcoma patients remain unclear and controversial. Our model showed a consistent treatment structure compared with the AJCC stage, and the results of both supported the idea that patients with different risk levels differ in the efficacy of adjuvant treatments. Consistent with previous findings, adjuvant treatment should be considered for patients concerned about residual lesions and those who were characterized for their elderly age, higher grade, larger tumor size, axial location, and advanced extension and considered a higher risk level based on the model (1). However, for extremely high-risk patients (nomogram stage V and AJCC stage IV), the results showed that the administration of adjuvant treatment had comparable prognoses to those of patients who did not receive adjuvant treatment. This result is consistent with some reports that chemotherapy failed to improve survival in the general advanced or metastatic synovial sarcoma patient population (29), although some studies have reported that chemotherapy increased the survival rate in advanced synovial sarcoma patients who experienced a good therapeutic response (30). Our model is limited by a lack of considering the influence of the therapeutic response, such as the histological response or the imaginal response, which weakens its ability to identify potential beneficiaries of chemotherapy and radiotherapy among advanced synovial sarcoma patients. Further improvement of the model could focus on expanding the treatment-related candidate variables predicting survival.

Our model could act as a supplementary tool for AJCC staging to use with synovial sarcoma patients. Compared with TNM-based AJCC staging, our nomogram-based risk stratification covers more synovial sarcoma-specific variables and smoothly qualifies the risk level in synovial sarcoma patients. Furthermore, our tool adjusts and redistributes the AJCC stage based on more synovial sarcoma-specific factors, including site, age, and gender. Regarding the treatment strategy, our model showed results consistent with those of the AJCC stage. AJCC stages II and III are comparable to the nomogram stage II to IV patient groups, who benefited from the adjuvant treatment including radiotherapy, chemotherapy, and the combination. As the results show, one advantage of our model is its ability to subdivide the low-risk patients, whose nomogram stage I patients had a 100.0% 5-year CSS subgroup within the AJCC stage II group with few risk factors and no need for additional adjuvant treatment. Our model also subdivided the nomogram stage II patients, who were beneficiaries of a single adjuvant treatment with only radiotherapy or chemotherapy when the AJCC II stage is relatively close to the nomogram stage III patients appeared to have greater survival rates with a combination of radiotherapy and chemotherapy.

Based on this model, a hypothetical 43-year-old female patient with a primary high-grade synovial sarcoma localized on the extremity with a d_max_ of 5.2 cm had a total score of 92.19. As shown in Figure 1, the corresponding predicted 5-year CSS rates were ~80–85%. Thus, this patient had an intermediate risk and needed to consider surgical resection and adjuvant treatment with a combination of radiotherapy and chemotherapy.

Our model has several limitations. First, the nomogram was validated with homogenous cases from the SEER database. This weakens the validation of the effectiveness of the model, and further prospective investigation is needed to quantify the effectiveness of the nomogram and the risk-adapted treatment strategy. Second, our model involves stratification based on the nomogram score; it lacks consideration of the correlations among different treatment protocols and clinical features, such as the various chemotherapy regimens, radiotherapeutic doses, and individual patient situations, including histological response, surgical resection margin, and baseline renal and hepatic function. Furthermore, since these confounding factors could have adverse effects on the prognostic implications of treatments, the clinical utility of the model could be limited. Further improvement of the model could focus on adding more complete variables regarding treatment protocols and patient clinical factors, in addition to using a cluster analysis to discover the actual cluster characteristics of synovial sarcoma patients and random decision forests and establish a model that can be used to guide the personalization of multimodal treatment based on patients' clinical feature clusters.

## Conclusion

In our study, we established a preoperative nomogram model to predict the 5-year CSS rate for patients with synovial sarcoma. The model combines independent prognostic predictors, including sex, age, grade, tumor location, size, and the EOD. Our nomogram had a high discriminatory ability and calibration for the prediction of 5-year CSS in both the training and validation groups. Based on this model, we assessed individual risk in the population and were able to provide a useful stratifying tool for multimodal risk-adapted adjuvant treatment in synovial sarcoma patients. Further improvement of the model could focus on a cluster analysis and include more personal clinical features to guide treatment selection.

## Data Availability Statement

Publicly available datasets were analyzed in this study. The datasets for this study can be found in the SEER database. The statistical analysis during the current study are available from the corresponding author on reasonable request.

## Ethics Statement

The studies involving human participants were reviewed and approved by Ethical Committee of the First Affiliated Hospital of Sun Yat-sen University. The patients/participants provided their written informed consent to participate in this study.

## Author Contributions

XX and LW designed the conception of the studies. ZZ and HY designed the research process and were major contributors in writing the manuscript. DL and QJ collected and assembled the data in SEER database. YB, YZ, JT, and BW participated in software support and data analysis. All authors read and approved the final manuscript.

## Conflict of Interest

The authors declare that the research was conducted in the absence of any commercial or financial relationships that could be construed as a potential conflict of interest.

## Abbreviations

STS: Soft tissue sarcoma
OS: Overall survival
CSS: Cancer-specific survival
H–L test: Hosmer–Lemeshow of fit test
AJCC: American Joint Committee on Cancer
SEER database: Surveillance, Epidemiology and End Results (SEER) database
t-ROC curve: time-dependent receiver operating characteristic curve
c-index: concordance index
CI: confidence intervals
KM method: Kaplan–Meier method.
