# Supplementary material for: Multimodal Risk-Adapted Treatment in Surgical Patients With Synovial Sarcoma: A Preoperative Nomogram-Guided Adjuvant Treatment Strategy
Source: Front Surg. 2020 Dec 21;7:579726. doi: 10.3389/fsurg.2020.579726 (PMC7780852; doi:10.3389/fsurg.2020.579726)

Supplementary File

**Supplementary Figure S1** Flowchart of establishment of nomogram model and risk-adapted therapy strategy


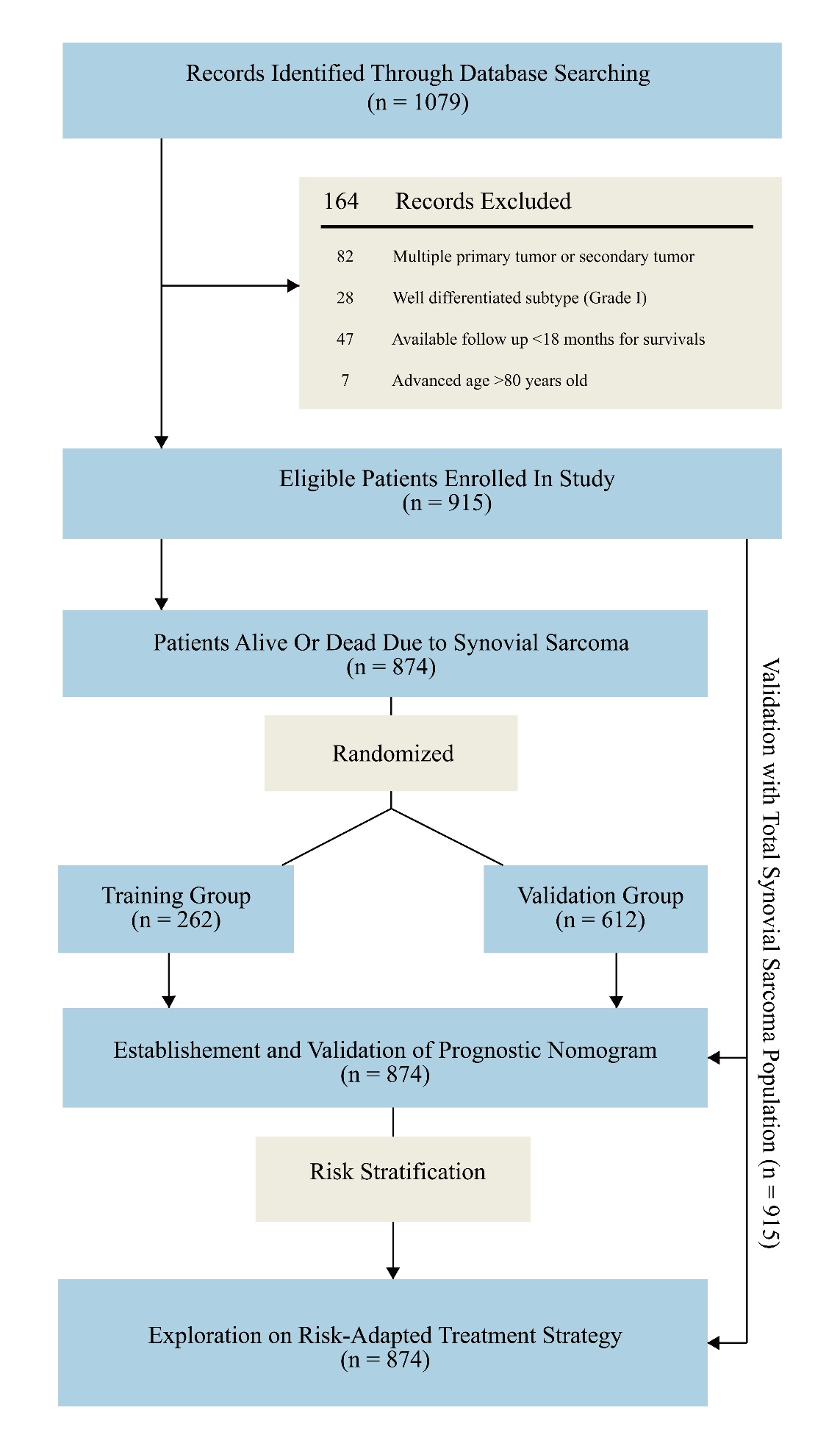


**Supplementary Figure S2** Annual numbers of newly diagnosed and dead synovial sarcoma during 1996-2015


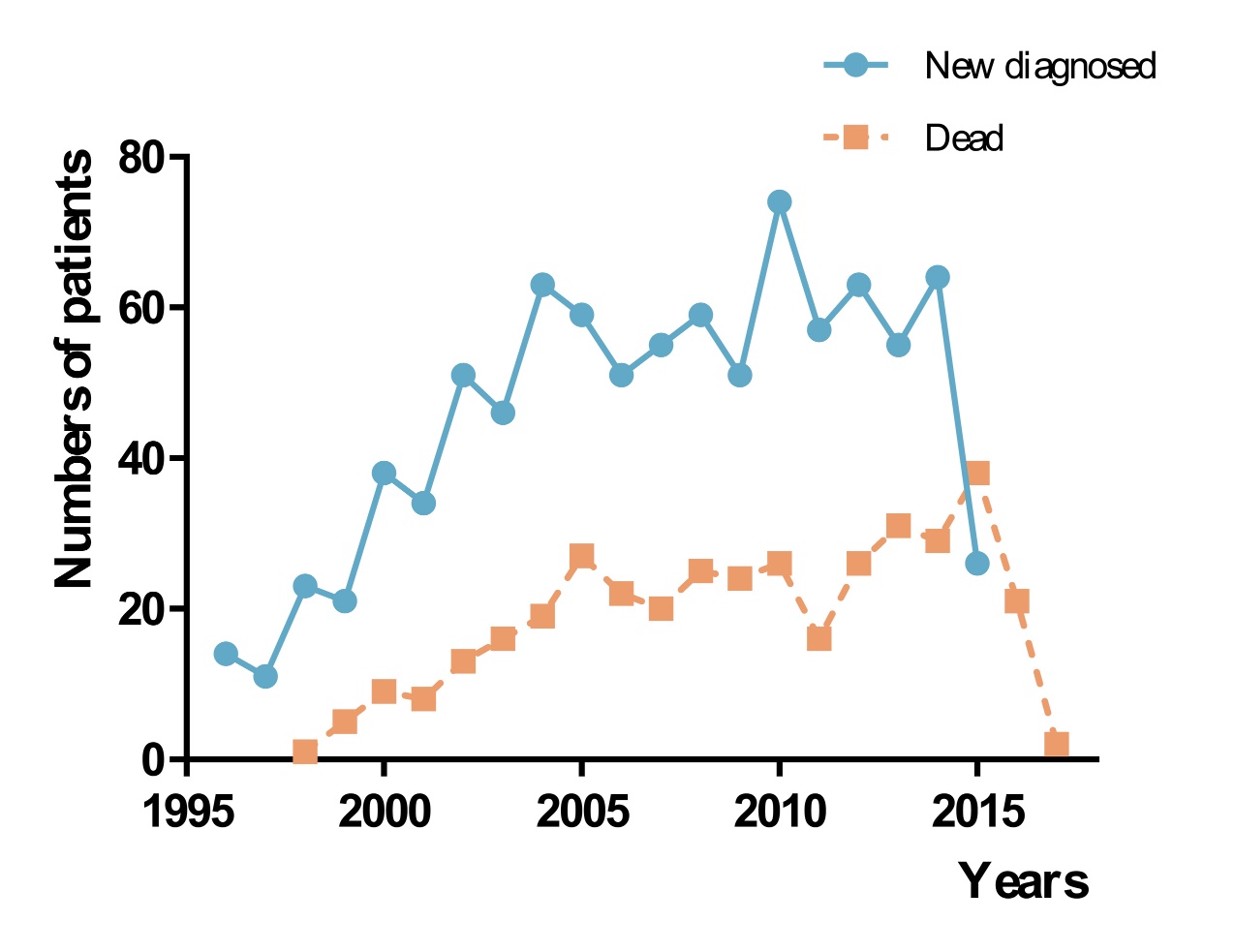


**Supplementary Figure S3** The estimated spline function of the age (A) and tumor size (B) on 5-year CSS rate.


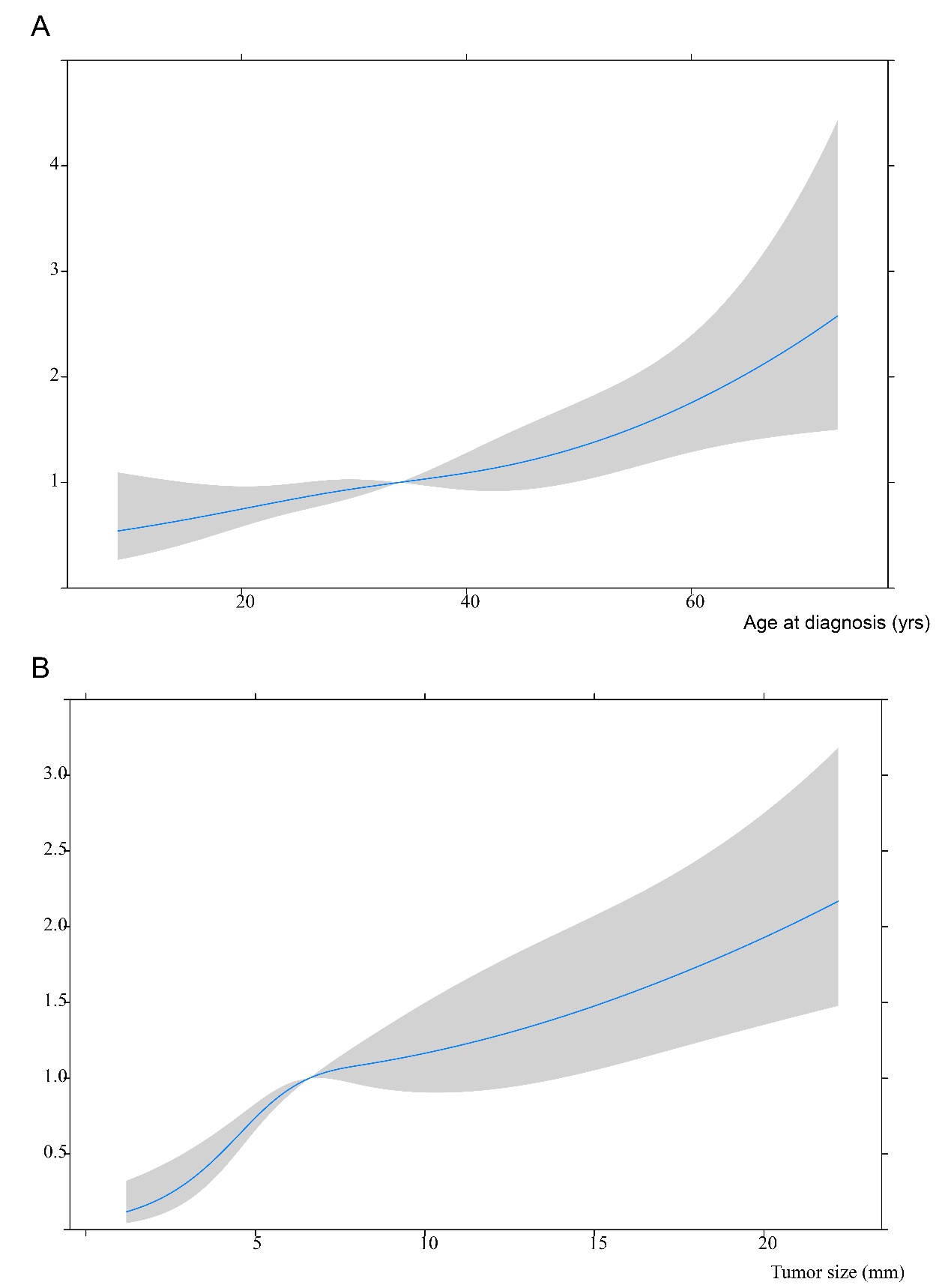


**Supplementary Figure S4** KM survival curves of enrolled categorical variable in the nomogram models

**A**: different genders; **B**: differentiate tumor locations; **C**: different grades; **D**: different extend of disease.


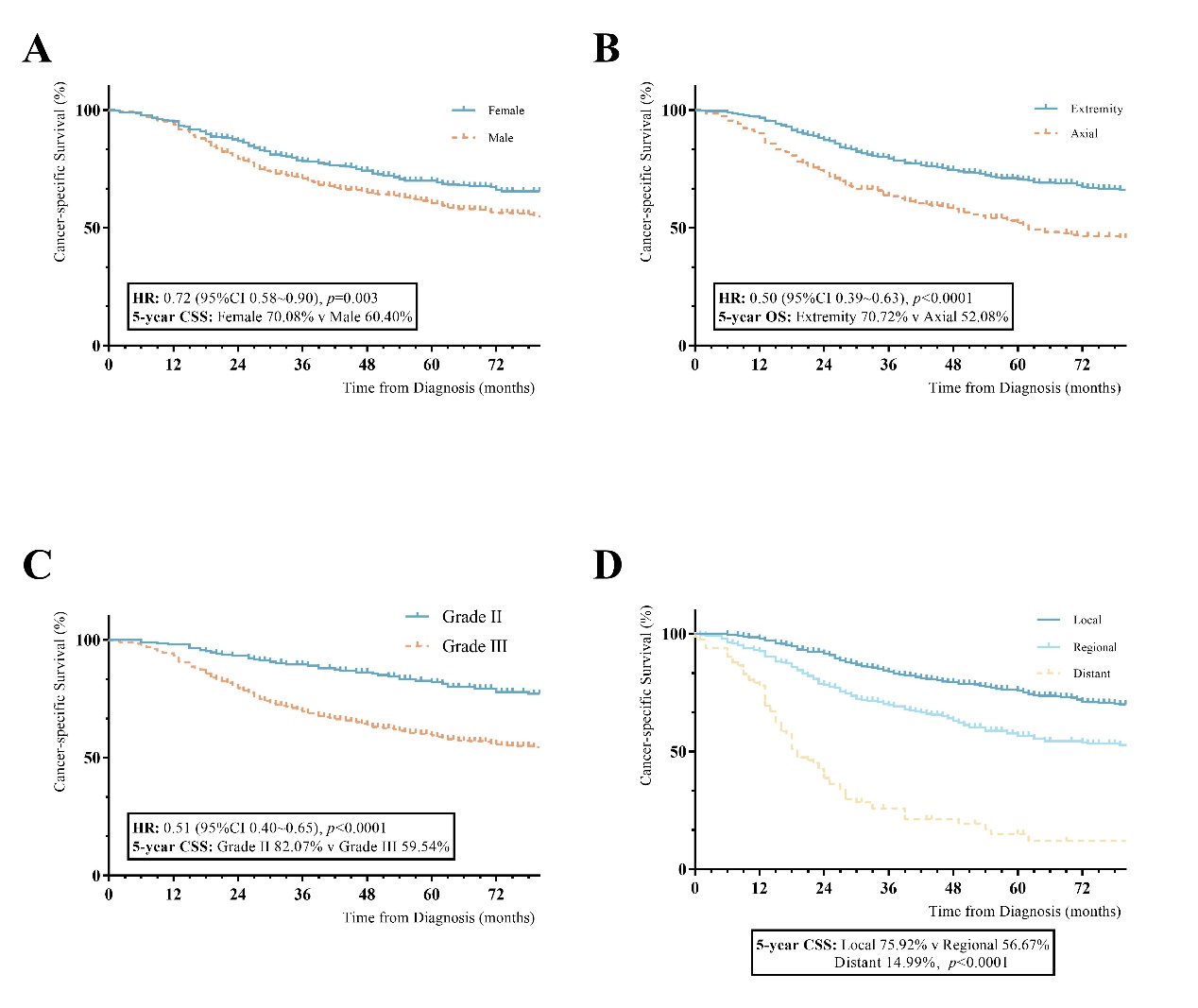


**Supplementary Figure S5** KM plot comparing the CSS difference among different risk groups


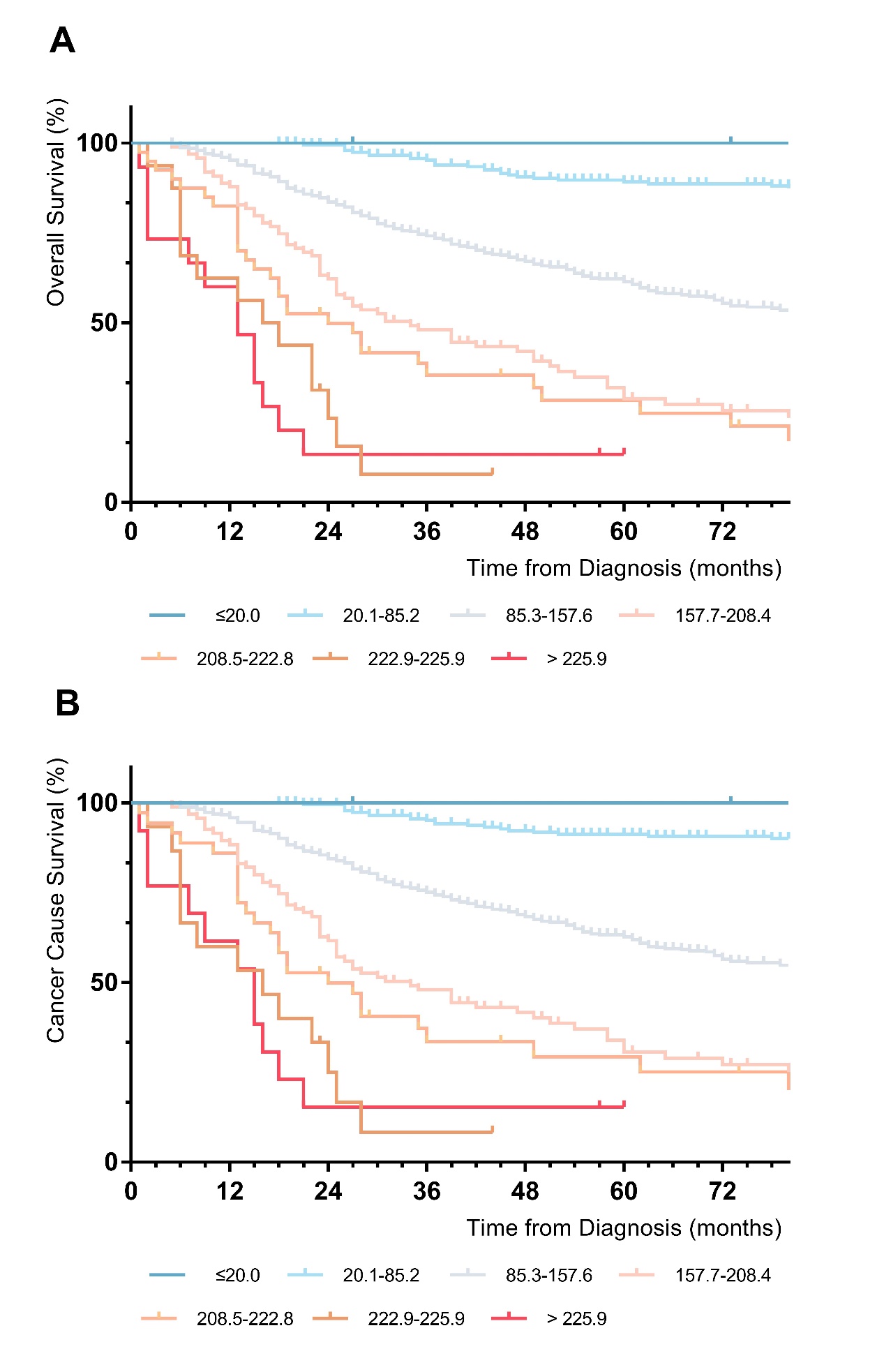

Supplement: Supplementary file 1 [file Table_1.DOCX]
